# Abl kinases can function as suppressors of tumor progression and metastasis

**DOI:** 10.3389/fonc.2023.1241056

**Published:** 2023-09-08

**Authors:** Melissa A. Marchal, Devon L. Moose, Afshin Varzavand, Nicole E. Jordan, Destiney Taylor, Munir R. Tanas, James A. Brown, Michael D. Henry, Christopher S. Stipp

**Affiliations:** ^1^ Department of Biology, College of Liberal Arts and Sciences, University of Iowa, Iowa City, IA, United States; ^2^ Department of Molecular Physiology & Biophysics, Carver College of Medicine, University of Iowa, Iowa City, IA, United States; ^3^ Department of Pathology, Carver College of Medicine, University of Iowa, Iowa City, IA, United States; ^4^ Holden Comprehensive Cancer Center, Carver College of Medicine, University of Iowa, Iowa City, IA, United States; ^5^ Department of Urology, Carver College of Medicine, University of Iowa, Iowa City, IA, United States

**Keywords:** ABL1, ABL2, prostate cancer, metastasis, cell motility, AKT, imatinib, extracellular matrix

## Abstract

**Introduction:**

Abl family kinases function as proto-oncogenes in various leukemias, and pro-tumor functions have been discovered for Abl kinases in many solid tumors as well. However, a growing body of evidence indicates that Abl kinases can function to suppress tumor cell proliferation and motility and tumor growth *in vivo* in some settings.

**Methods:**

To investigate the role of Abl kinases in tumor progression, we used RNAi to generate Abl-deficient cells in a model of androgen receptor-indifferent, metastatic prostate cancer. The effect of Abl kinase depletion on tumor progression and metastasis was studied in an *in vivo* orthotopic model, and tumor cell motility, 3D growth, and signaling was studied *in vitro*.

**Results:**

Reduced Abl family kinase expression resulted in a highly aggressive, metastatic phenotype *in vivo* that was associated with AKT pathway activation, increased growth on 3D collagen matrix, and enhanced cell motility *in vitro*. Inhibiting AKT pathway signaling abolished the increased 3D growth of Abl-deficient cells, while treatment with the Abl kinase inhibitor, imatinib, promoted 3D growth of multiple additional tumor cell types. Moreover, Abl kinase inhibition also promoted soft-agar colony formation by pre-malignant fibroblasts.

**Conclusions:**

Collectively, our data reveal that Abl family kinases can function to suppress malignant cell phenotypes *in vitro*, and tumor progression and metastasis *in vivo*.

## Introduction

Abelson murine leukemia viral oncogene homolog 1 (*ABL1*) was first identified over 50 years ago as the transforming gene present in Abelson murine leukemia virus (1). The viral oncoprotein, v-Abl, was shown to exhibit tyrosine kinase activity (2, 3) and to be homologous with mouse cellular Abl (4). The human ortholog of Abl was later mapped to the long arm (q) of chromosome 9 (5) and identified as part of the constitutively active BCR-ABL fusion protein (6). BCR-ABL is localized to the Philadelphia chromosome, which is generated by the reciprocal chromosomal translocation t(9;22)(q34;q11) (7), and is the major oncogenic driver in Philadelphia-positive (Ph+) leukemia cells. *ABL2*, also known as Abl related gene (Arg), is paralogous with *ABL1* and was subsequently discovered by sequence similarity (8, 9).

Much of the earlier literature describing the signaling functions of Abl kinases in cancer has been shaped by studies of leukemia cells that focused on the biology of BCR-ABL fusion proteins and less common fusions involving ABL2. Although several studies identified pro-apoptotic functions for ABL1 in response to DNA damage (10–13), in solid tumors, Abl kinases are widely regarded as promoters of tumor progression and metastasis (14, 15). Nevertheless, a growing number of studies indicates that Abl kinases can function to restrain malignant behavior in multiple settings, under circumstances that do not necessarily involve a response to overt DNA damage (16–20). As with Abl kinases themselves, Abl kinase substrates such as Crk and ABI1 may have context-dependent tumor promoting or tumor suppressor functions (21–24). We previously identified Abl kinases as potential negative regulators of malignant behaviors operating downstream of α3 integrin in a model of metastatic prostate cancer (25). However, the role of Abl kinases in progression and metastasis *in vivo* was not determined, and the molecular mechanism by which Abl kinases could function to restrain malignant behaviors remained to be fully defined. We now find that loss of Abl kinase expression can dramatically potentiate progression and metastasis *in vivo*, which corresponds to sustained AKT signaling and tumor cell growth on 3D matrix, and strikingly increased tumor cell motility. Our results reveal a novel mechanism by which Abl kinases can function to restrain the malignant behaviors of tumor cells and inhibit tumor progression and metastasis *in vivo*.

## Materials and methods

### Cell lines and culture

GS689.Li cells are a metastatic variant of PC-3 prostate cancer cells, created by *in vivo* passaging PC-3 prostate cancer cells in SCID mice (26), and reverified by STR analysis (IDEXX Bioresearch) (25). Other cell lines used in this study were obtained directly from ATCC. Base growth media and supplements were from Gibco ThermoFisher. GS689.Li cells and DU-145 cells were cultured in DMEM:F12, 22Rv1, LNCaP, Caov-3, OVCAR-3 and Renca cells were cultured in RPMI, and A2058, A375, MDA-MB-468, MDA-MB-231, and VCaP cells were cultured in DMEM. Growth media were supplemented glutamine, penicillin/streptomycin, non-essential amino acids, and 10% FBS (Atlanta Biologicals). All cell lines were stably transduced to express luciferase using the pQCXIN retroviral vector followed by selection with G418.

### Antibodies and reagents

This study utilized antibodies from Cell Signaling Technology against FOXA2 (#8186), Rb (#9309), c-ABL (#2862), FAK (#3285), SRC (#2110), phospho-SRC Tyr416 (#6943), ERK (#4696), phospho-ERK (#9101), phospho-AKT Ser473 (#4060), phospho-AMPKα Thr172 (#2535), Cyclin D3 (#2936), phospho-S6 Ribosomal Protein Ser240/244 (#5364) and YAP/TAZ (#8418). Also used were antibodies from BD Transduction against phospho-FAK Tyr397 (#611806), and AKT (#610860). Additional primary antibodies were α-tubulin (12G10, Developmental Studies Hybridoma Bank), and ARG (Arg 11, provided by Anthony Koleske, PhD, Yale University). Secondary antibodies used were goat-anti-mouse and goat-anti-rabbit antibodies conjugated to Alexa 790 or 680 (Invitrogen). Other reagents were D-luciferin (Gold Biotechnology), rat tail collagen I (Corning & Advanced Biomatrix), imatinib (Cayman Chemical), GNF5, MK-2206 (Selleckchem) and the small molecule Abl kinase activator, DPH (Millipore Sigma).

### RNA interference

The specificity and efficacy of ABL and ARG knockdown was previously validated by multiple independent RNAi targeting vectors (25). We used the most effective RNAi vectors from our previous study to create Abl (ABL KD) and Arg (ARG KD) single and double knockdown (ABL/ARG KD) GS689.Li cells. The Abl sh3 retroviral construct has a pZIP-mCMV-ZsGreen backbone (Transomics Technologies) and the Arg sh2 retroviral construct has a pSIREN RetroQ vector backbone (Clontech).

All four cell lines were carefully matched by passage number and contained both vector backbones. Thus, vector controls (NT/NT) contained both non-targeting shRNAs in the corresponding vector backbones, single knockdowns contained one targeting shRNA in one vector backbone and one non-targeting shRNA in the other vector backbone, and double knockdowns contained both targeting shRNAs in the corresponding vector backbones. Because each cell line in the set contained a pZIP-mCMV-ZsGreen vector, all cells were fluorescently labeled. Cells were maintained as stably transduced, polyclonal populations. The RNAi targeting sequences used for the present study were as follows: Abl sh3, 5’-GCAGTCATGAAAGAGATCAAA-3’ (ULTRA-3267084, Transomics); and Arg sh2, 5’-CCTCAAACTCGCAACAAAT-3’.

### Orthotopic prostate cancer model

All animal protocols were approved by the University of Iowa Institutional Animal Care and Use Committee (Approval #8031328). On the day of inoculation, 5 X 10^4^ NT/NT, ABL KD, ARG KD, and ABL/ARG KD GS689.Li cells were implanted in the left or right anterior lobe of the prostate of 10 SCID/NCr (BALB/C) mice/cell line. Bioluminescent imaging (BLI) was performed using an Ami X imaging system (Spectral Instruments Imaging) as described previously (25). Upon sacrifice, livers, kidneys, and lungs were dissected for analysis of disseminated cells by fluorescence microscopy using an Olympus SZX12 stereomicroscope, as previously described (25). GS689.Li cells from primary tumors were recovered by mincing with a sterile razor blade and digesting with 200 U/ml collagenase II (Worthington Biochemical Corporation) for 15 min at 37°C. Explanted cells were grown out under G418 to select for luciferase-positive tumor cells.

### Time-lapse motility assays

A total of 2.3 x 10^5^ cells were plated in serum-free medium [SFM; DMEM:F12, 5 mg/mL BSA, 2 mmol/L L-glutamine, 100 U/mL penicillin,100 mg/mL streptomycin, 0.1 mmol/L nonessential amino acids, 25 mmol/L HEPES pH 7.2, plus insulin–transferrin–selenium (ITS) supplement] on 35-mm dishes coated with 10 ug/mL rat tail collagen I. Time-lapse images were acquired as described previously (25), and migration speed and displacement were calculated using the mTrackJ plugin (27). ImageJ software was used to measure the morphological characteristics of migrating cells (at t = 20 minutes) by making cell traces using the Freehand selections tool, followed by the Measure (Shape descriptors) command.

### 3D collagen growth assays

Twenty-four-well plates were coated with 350 µL of rat tail collagen I (0.8 mg/mL in DMEM) per well and allowed to polymerize for 45 minutes at 37°C. A total of 1.5x10^4^ cells per well were seeded in 500 µL of SFM. After 7–10 days, cell number was quantified by BLI. Six wells per cell type per condition were quantified in each trial.

### Standard growth assays

A total of 1.5x10^4^ cells per well were plated in uncoated 24-well plates in 500 µL of standard growth medium. After 4–7 days, cell number was quantified by BLI. Six wells per cell type were quantified in each trial.

### Reverse phase protein array

For reverse phase protein array (RPPA) analysis, cells growing on 3D collagen were lysed in 10% glycerol, 2% SDS, 0.0625 M Tris‐HCL, pH 6.8, and 2.5% beta‐mercaptoethanol, and lysate concentrations were adjusted to 1.0 ug/uL. Duplicate lysates of NT/NT and ABL/ARG KD cells were analyzed at the Functional Proteomics RPPA core facility (MD Anderson Cancer Center). Normalized linear protein levels for each of 304 signaling proteins included in the analysis were used to calculate the normalized linear fold change [(normalized linear value ABL/ARG KD)/(normalized linear value NT/NT)], and a cutoff was set for an average fold change of 1.2 or greater or 0.8 or less. A heatmap was generated using median centered log_2_ values from targets meeting the cutoff criteria, using the Heatmapper tool (http://www.heatmapper.ca/).

### Immunoblotting

Cells were rinsed twice with HBSM and lysed by scraping into Laemmli buffer. For analysis of cells growing in 3D conditions, cells growing on 0.8 mg/ml collagen I were rinsed twice with HBSM and then incubated two minutes at room temperature (RT) with Laemmli buffer. Lysate was collected from the surface of the 3D matrix by pipetting. Protein concentrations of lysates were normalized using the RED 660 Protein Assay (G Biosciences) prior to SDS-PAGE and immunoblotting. Blots were analyzed using a LiCOR Odyssey blot imager.

### Soft agar growth assays

Indicated numbers of NIH-3T3 cells were plated in a top layer of 0.35% agarose over a bottom layer of 0.5% agarose in DMEM with 10% FBS in 6 well plates, and overlaid with 1 ml of growth medium containing 10% FBS. DMSO vehicle or 2µM Abl kinase inhibitor GNF5 were added to a to the agarose layers and the overlay. Cultures were refed once per week with fresh growth medium containing DMSO or GNF5, and then photographed for colony counting after 3 weeks of growth.

## Results

### Abl kinases can function to suppress tumor progression and metastasis *in vivo*

**Figure 1:**
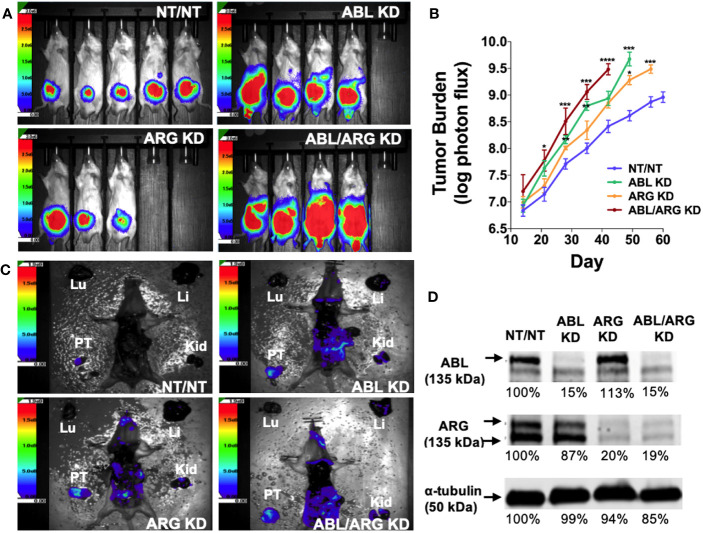
Loss of Abl family kinase expression promotes tumor progression and metastatic dissemination. **(A)** Representative bioluminescence (BLI) images of orthotopic tumors in mice implanted with Abl family kinase-deficient (ABL KD, ARG KD, and ABL/ARG KD) and non-targeting (NT/NT) tumor cells at 35 days post-implantation. **(B)** Semi-log plot depicting average tumor burden (log of photon flux [photons/s]) over time (day) for mice bearing NT/NT (blue line), ABL KD (green line), ARG KD (orange line) and ABL/ARG KD (red line) tumors. Data points indicate mean +/- SEM for ≥ 7 mice/cell type. *, **, ***, and **** signify statistically significant p-values of < 0.05, < 0.01, < 0.001, and < 0.0001, respectively. Kruskal Wallis with Dunn’s multiple comparison test and Mann Whitney test at α = 0.05. **(C)** Representative images of BLI-assisted gross necropsies performed immediately after sacrifice for mice harboring NT/NT (sacrificed day 63), ABL KD (sacrificed day 52), ARG KD (sacrificed day 59), and ABL/ARG KD (sacrificed day 45) tumors. PT: primary tumor; Kid: kidney; Li: liver; Lu: lung. **(D)** Immunoblot analysis of ABL and ARG proteins in all four cell types prior to implantation. % values represent blot intensities normalized by an α-tubulin loading control and expressed as percent relative to NT/NT. Color scales in **(A, C)** indicate photons/sec/cm^2^/sr.

To investigate the role of the Abl family kinases in tumor progression and metastasis, we depleted ABL1 (ABL KD), ABL2 (ARG KD) or both kinases (ABL/ARG KD) in GS689.Li cells, a model of spontaneously metastatic prostate cancer (26). Luciferase/ZsGreen dual-labeled, Abl kinase-deficient and non-targeting control cells (NT/NT), were orthotopically implanted, and *in vivo* tumor progression was monitored weekly via BLI. By day 35 after implantation, mice harboring ABL KD cells and ABL/ARG KD cells displayed a markedly higher apparent tumor burden than mice harboring ARG KD or NT/NT control cells (**Figure 1A**). Across the course of the experiment, Abl kinase-deficient tumors progressed more rapidly than control tumors (**Figure 1B**). Loss of ABL had a large effect, but loss of both ABL and ARG had the largest effect, resulting in tumor burdens over an order of magnitude greater than NT/NT control tumors by day 35.

To attempt to control for effects of primary tumor size, we allowed mice in the different groups to age out for different times until their total tumor burdens approached a similar value, although the vector control group still had lower average tumor burden at the time of sacrifice. Shown in **Figure 1C** are gross necropsy images of mice with NT/NT control, ABL KD, ARG KD, and ABL/ARG KD tumors. The apparent tumor burden of all four mice was ~1 X 10^9^ photons/sec prior to sacrifice, yet the dissemination of Abl kinase-deficient tumor cells appeared qualitatively substantially increased compared to vector control tumor cells (**Figure 1C**), despite the fact that the mice harboring vector control tumor cells were euthanized ~2 additional weeks later than the mice harboring the Abl-deficient tumor cells. This qualitative example motivated us to further study tumor progression in mice harboring control versus Abl-deficient cells. Analysis of tumor cells explanted at the time of sacrifice revealed an 80-85% reduction in Abl and Arg proteins (**Figure S1**), which was similar to what we observed in ABL and ARG KD cells prior to implantation (**Figure 1D**), demonstrating that ABL and ARG knockdown was maintained in tumor cells *in vivo*.

**Figure 2:**
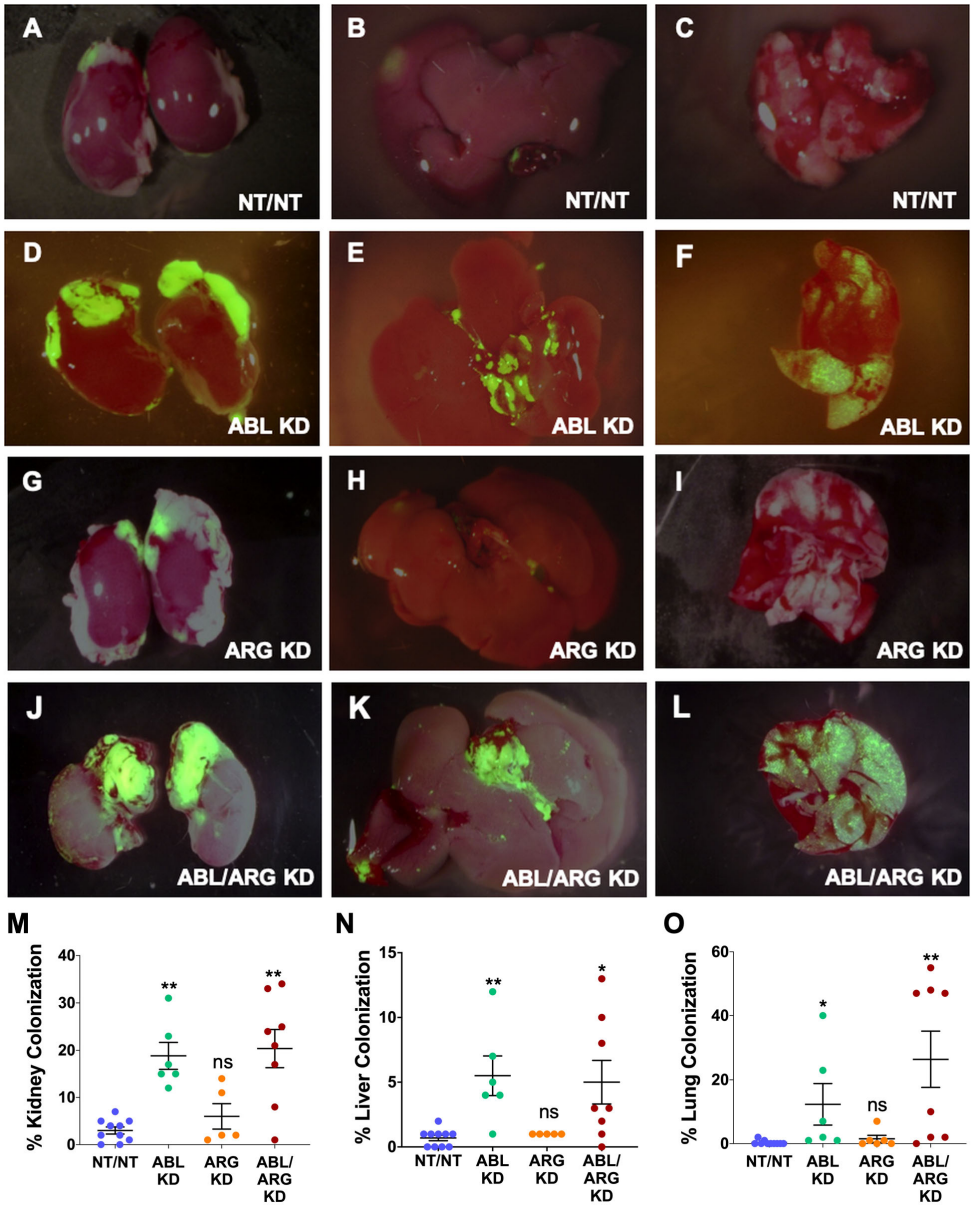
Abl kinase-deficient tumor cells exhibit enhanced metastatic colonization. Representative fluorescent micrographs of kidneys (1^st^ column), livers (2^nd^ column), and lungs (3^rd^ column) colonized by GFP-labeled NT/NT **(A–C)**, ABL KD **(D–F)**, ARG KD **(G–I)**, and ABL/ARG KD **(J–L)** tumor cells. Quantification of kidney **(M)**, liver **(N)**, and lung **(O)** colonization as the percentage of total surface area occupied by GFP-labeled tumor cells. Bars in **(M–O)** indicate mean +/- SEM for ≥ 5 organs/cell type. * and ** in **(M-O)** denote statistically significant p-values of < 0.05 and < 0.01, respectively, and n.s, stands for not significant. Kruskal-Wallis with Dunn’s multiple comparison test, α = 0.05.

We used ZSGreen fluorescence microscopy to further investigate tumor cell dissemination to specific organs harvested at the time of sacrifice. ABL KD and ABL/ARG KD tumor cells both displayed significantly increased dissemination to kidney (primarily in the adjacent adrenal gland), liver, and lung compared to vector control cells (**Figures 2A–L**, quantified in **M–O**). For ARG KD single knockdown cells, there was a trend towards increased kidney colonization, although it did not rise to the level of statistical significance in this analysis (**Figure 2M**).

**Figure 3:**
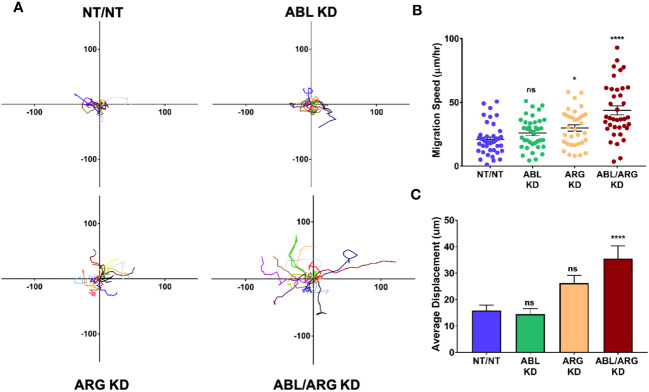
Abl-deficient tumor cells display increased migration velocity. Abl family kinase-deficient (ABL KD, ARG KD, and ABL/ARG KD) and non-targeting (NT/NT) tumor cells were plated on 2D collagen I and had their migration monitored via time-lapse microcopy for 90 minutes. **(A)** Motility tracks; n = 20 tracks/cell type. X/Y axes in um, 0 represents point of origin. **(B)** Graph of average migration speed (um/hr.), n = > 34 cells/cell type. **(C)** Graph of average displacement (um) from point of origin, n = > 34 cells/cell type. Bars in B & C indicate mean +/- SEM. * and **** in B & C indicate statistically significant p-values of < 0.05 and < 0.0001, respectively, and n.s. stands for not significant. One-way ANOVA w/Dunnett’s multiple comparison, α = 0.05.

Because it is difficult to fully account for the contribution of more rapid progression of the primary tumor to increased metastatic dissemination, we also compared the motility of Abl-deficient tumor cells to vector control cells via time lapse video microscopy. ABL/ARG KD cells displayed dramatically increased motility compared to vector control cells, as visualized by cell motility tracks (**Figure 3A**), with ABL KD and ARG KD single knockdown cells displaying an intermediate phenotype. Quantification revealed a striking ~2-fold increase in migration speed and net displacement of ABL/ARG KD cells compared to vector control cells, with ARG single knockdown producing a larger effect than ABL single knockdown on cell motility. (**Figures 3B, C**). The enhanced motility of Abl kinase-deficient cells was associated with a morphological change from round, spread vector control cells to highly polarized Abl kinase-deficient cells, with prominent leading edge lamellipodia (**Figures S2A–D**). This morphological change is quantitatively captured as a significant increase in the aspect ratio of ARG KD and ABL/ARG KD cells (**Figure S2E**), and a significant reduction in roundness (**Figure S2F**).

Taken together, these data revealed Abl kinases can function as suppressors of progression and metastatic dissemination *in vivo*, restraining tumor growth, metastatic colonization, and cell motility. Loss of ABL had a larger effect on tumor growth, while loss of ARG had a larger effect on cell motility. These observations may help to explain why loss of both kinases produced the most aggressive phenotype in the *in vivo* tumor progression model.

### Abl kinase-deficient tumor cells show enhanced growth on 3D matrix associated with sustained AKT signaling

**Figure 4:**
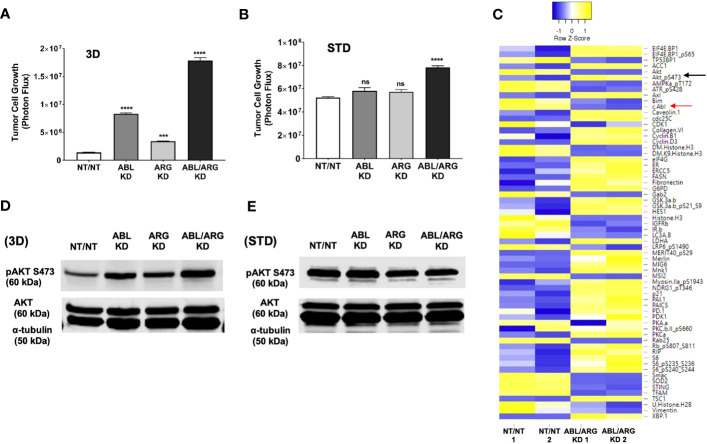
Increased 3D growth and AKT pathway activation of Abl-deficient tumor cells. Graphs of tumor cell growth (photon flux [photons/s]) for Abl family kinase-deficient (ABL KD, ARG KD, ABL/ARG KD) and non-targeting (NT/NT) tumor cells growing under **(A)** 3D or **(B)** standard (STD) conditions for seven days. Bars in A & B indicate mean +/- SEM for 6 wells/cell type/condition. *** and **** denote statistically significant p-values of < 0.001 and < 0.0001, respectively, and n.s. stands for not significant. One-way ANOVA with Sidak multiple comparison test, α = 0.05. **(C)** Heatmap displaying median-centered, normalized log_2_ values of differentially expressed antigens (DEAs) identified by RPPA. Lysates of NT/NT and ABL/ARG KD mCRP tumor C cells grown under 3D conditions for 4 days were prepared in duplicate for analysis by RPPA. Antigens with average normalized linear fold changes > 1.2 or < 0.8 were considered DEAs. Rows corresponding to pAKT S473 (black arrow) and c-Abl (red arrow) are indicated. (**D, E**) Immunoblot analysis of AKT phosphorylated on serine 473 (pAKT S473), total AKT (AKT), and an α–tubulin loading control for Abl family kinase-deficient and NT/NT control tumor cells growing under **(D)** 3D or **(E)** STD conditions for 4 days.

To establish a system to investigate the molecular basis of increased tumor growth of Abl kinase-deficient cells, we examined low anchorage growth on a soft 3D collagen matrix in defined medium, an assay that has aligned well with the *in vivo* behavior in our model of prostate cancer metastasis (25). Similar to the tumor growth *in vivo*, ABL/ARG KD cells displayed dramatically enhanced 3D growth compared to vector control cells, with ABL single knockdown producing a larger effect than ARG single knockdown (**Figure 4A**). These differences were context specific, and largely attenuated under standard tissue culture conditions (**Figure 4B**), although ABL/ARG KD still showed modestly increased proliferation compared to control cells.

To investigate changes in cell signaling that could account for the enhanced 3D growth of Abl-deficient cells, we generated lysates of NT/NT and ABL/ARG KD cells growing in 3D conditions for analysis by reverse phase protein array (RPPA). Returned normalized linear values were used to calculate average fold changes (normalized linear ABL/ARG KD/normalized linear NT/NT) across all 300+ RPPA antibodies. An arbitrary cut off was set for an average fold change of 1.2 or 0.8. Antibodies with average fold changes meeting the cutoff criteria were visualized by a heatmap using median centered normalized log_2_ values (**Figure 4C**; see **Table S1** for full results of the RPPA).

As expected, c-ABL (fold change = 0.7; red arrow **Figure 4C**) was among the 64 differentially expressed antigens (DEAs) we identified. Notably, activated AKT (pAKT S473) was upregulated in Abl-deficient cells compared to vector controls (fold change = 1.5; black arrow **Figure 4C**). In addition, several canonical AKT signaling components were also differentially expressed (selected list in **Figure S3A**). Pathway analysis with DAVID showed that 26.3% of DEAs were associated with the PI3K-AKT signaling pathway (P = 6.7x10^-8^). Cell cycle factors were also greatly enriched in DEAs (selected list in **Figure S3B**), with DAVID pathway analysis showing 15.8% being associated with the cell cycle (P = 2.2x10^-6^). Proteins involved in metabolism and lipogenesis (selected list in **Figure S3C**) were also prevalent among DEAs and 15.8% of them were associated with AMPK signaling (DAVID pathway analysis; P = 2.2x10^-6^).

Immunoblotting confirmed that Abl-deficient cells displayed a prominent increase in AKT activation, in a pattern that aligned with their 3D growth. ABL/ARG KD cells had the highest level of AKT activation, followed by ABL single knockdown cells and ARG single knockdown cells (**Figure 4D**). Quantification of activated pAKT S473 revealed an ~3.9-fold increase in ABL/ARG KD cells, and ~2.2-fold and ~1.4-fold increase in ABL and ARG single knockdown cells respectively, relative to NT/NT vector control cells growing on 3D collagen. Importantly, under standard tissue culture conditions, all four cell lines displayed similar levels of AKT activation (**Figure 4E**), consistent with their similar growth under those conditions (**Figure 4B**). Thus, the regulatory relationship between Abl kinase expression and AKT activation is context-dependent. All four cell types have strong AKT activation under standard 2D conditions, but Abl-deficient cells are better able to maintain AKT activation when placed on a 3D matrix in the absence of serum growth factors.

To further validate our RPPA data, we used immunoblotting to analyze signaling proteins from each of the categories identified during DAVID pathway analysis (**Figures S3D–F**). Expression of the canonical AKT signaling target S6 Ribosomal Protein phosphorylated at serine 240/244 (pS6 S240/244) (**Figure S3D**), and cyclin D3 (**Figure S3E**), mirrored the pattern of pAKT S473 expression in Abl family kinase-depleted cells, with the highest expression levels of these antigens in ABL/ARG KD double knockdown and ABL KD single knockdown cells. Conversely, expression of AMPKα phosphorylated at its activating threonine 172, a site negatively regulated by AKT (28), was downregulated in ABL/ARG KD cells (**Figure S3F**). Overall, our immunoblotting data further validated that loss of the Abl family kinases can promote AKT pathway activation in low-anchorage, 3D growth conditions. Changes in signaling status of ERK, FAK/Src, or YAP/TAZ signaling proteins were not detected in Abl-deficient cells, either by RPPA or by immunoblotting (**Figure S4**).

### Pharmacological inhibition of Abl kinase activity can promote a malignant phenotype

**Figure 5:**
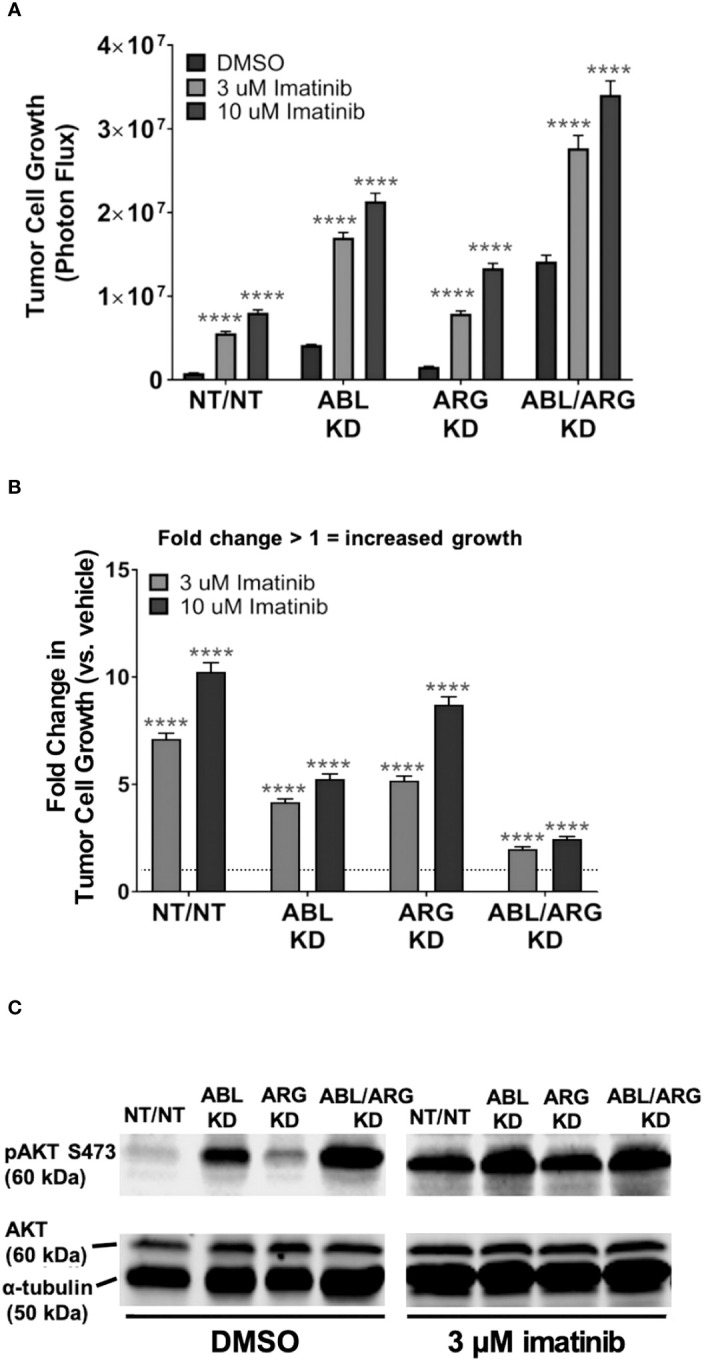
Pharmacological inhibition of Abl kinases can promote increased tumor cell 3D growth and AKT pathway activation. Graphs of **(A)** tumor cell growth (photon flux [photons/s]) and **(B)** fold change in tumor cell growth relative to vehicle for Abl family kinase-deficient (ABL KD, ARG KD, and ABL/ARG KD) and non-targeting (NT/NT) tumor cells growing in 3D and treated with DMSO (white), 3 uM imatinib (light gray), or 10 uM imatinib (black) for 7 days. Bars in **(A, B)** indicate mean +/- SEM for 6 wells/cell type/condition. In (A)**** indicates statistically significant p-values < 0.0001 within each cell type for a specific dose of imatinib relative to DMSO control. Unpaired t-test with Holm-Sidak correction for multiple t-tests, α = 0.05. In (B)*** and **** denote statistically significant p-values of < 0.001 and < 0.0001, respectively, for each Abl family kinase-deficient cell type relative to NT/NT cells for a specific dose of imatinib. Two-way ANOVA with Sidak multiple comparison test, α = 0.05. **(C)** Immunoblot analysis of AKT phosphorylated on serine 473 (pAKT S473), total AKT (AKT), and an α–tubulin loading control for Abl family kinase-deficient and non-targeting tumor cells growing in 3D and treated with DMSO or 3 uM imatinib for 4 days.

The data presented above suggested that the Abl kinase inhibitor imatinib, developed for targeted inhibition of the BCR-Abl fusion protein, might have paradoxical, pro-malignant effects in some contexts, in cells expressing non-mutant Abl kinases. However, Abl kinases also have scaffolding functions, and kinase-dead Abl mutants can rescue some phenotypes of Abl loss of function (29). We predicted that if loss of Abl kinase activity *per se* contributed to the increased pAKT S473 and 3D growth of Abl-deficient cells, then imatinib treatment should at least partially phenocopy genetic depletion of Abl kinases. When we treated cells growing in 3D with imatinib, we saw significant increases in growth for all cell types relative to their DMSO controls at both 3 uM and 10 uM imatinib, with the higher dose generating an even greater response (**Figure 5A**). The fact that even the higher dose of imatinib could not fully phenocopy the genetic depletion of Abl kinases when applied to NT/NT control cells is consistent with potential scaffolding functions for Abl kinases that may not be fully blocked by inhibiting kinase activity. Conversely, the fact that ABL/ARG KD cells still displayed increased 3D growth upon treatment with imatinib probably reflects inhibition of the kinase activity of the residual amounts of ABL (15%) and ARG (20%) remaining in these cells. The NT/NT cells experienced the greatest fold change in growth compared to all other cell types at both doses of imatinib, and ABL/ARG KD cells exhibited the smallest fold change in growth, with single knockdown cells displaying an intermediate phenotype (**Figure 5B**). In parallel with its ability to promote 3D growth, imatinib treatment dramatically increased phospho-AKT S473 in vector control cells and ARG single knockdown cells, but had minimal to no effect on ABL and ABL/ARG KD cells, in which phospho-AKT levels were already very high (**Figure 5C**). These data revealed that Abl kinase activity is an important contributor to the ability of Abl kinases to suppress 3D growth of tumor cells, but scaffolding functions might also contribute to suppressor functions.

**Figure 6:**
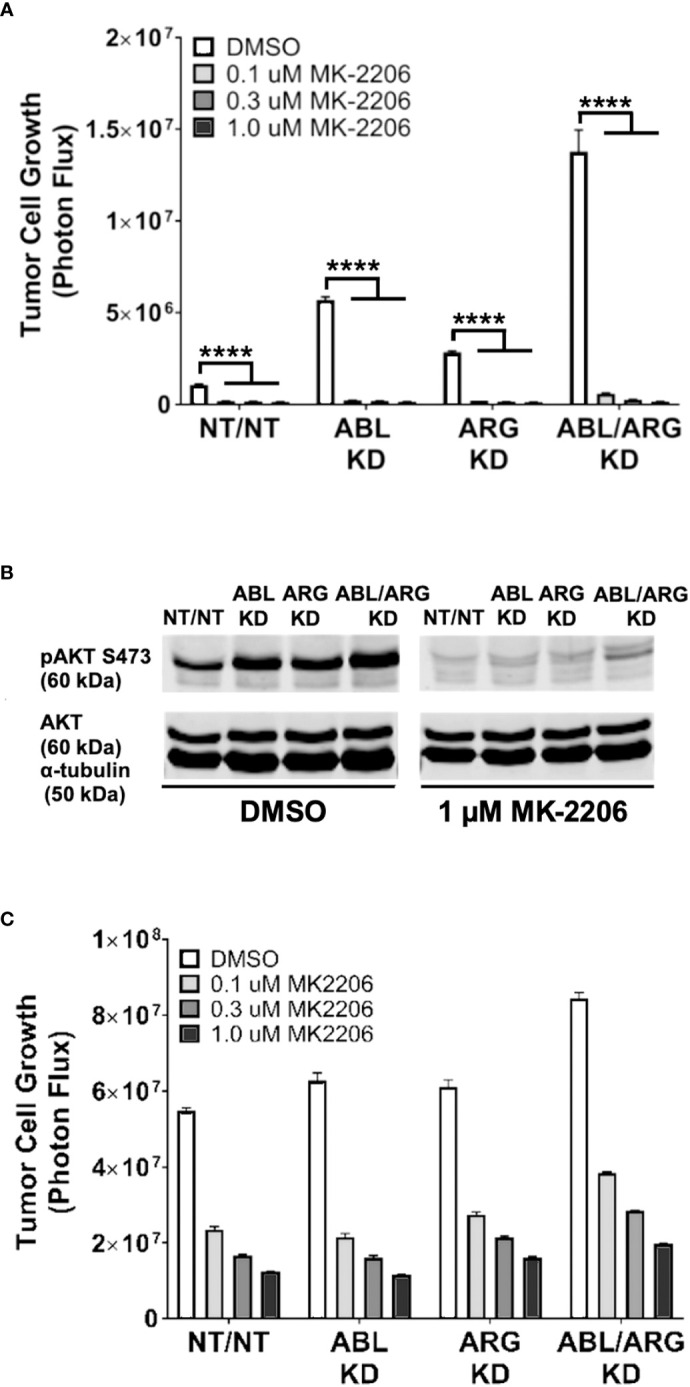
The AKT inhibitor, MK-2206, abolishes the increased 3D growth and AKT activation of Abl family kinase-deficient tumor cells. Graphs of tumor cell growth (photon flux in [photons/s]) for Abl family kinase-deficient (ABL KD, ARG KD, ABL/ARG KD) and non-targeting (NT/NT) tumor cells under 3D **(A)** and standard **(C)** conditions and treated with DMSO (white), 0.1 uM MK-2206 (light gray), 0.3 uM MK-2206 (dark gray), or 1.0 uM MK-2206 (black) for 7 days. Bars in **(A, C)** indicate mean +/- SEM for 6 wells/cell type/condition. **** in (A) signifies statistically significant p-values <0.0001. Unpaired t-test with Holm-Sidak correction for multiple t-tests, α = 0.05. **(B)** Immunoblot analysis of AKT phosphorylated on serine 473 (pAKT S473), total AKT (AKT), and an α–tubulin loading control for Abl family kinase-deficient and non-targeting tumor cells growing in 3D for 4 days and treated with DMSO or 1.0 uM MK-2206 for 1 day.

To determine the extent to which AKT signaling is required for the increased 3D growth of Abl kinase-deficient prostate cancer cells, we treated cells with increasing doses of the AKT inhibitor MK-2206. MK-2206 abolished the 3D growth of all 4 cell types, even the ABL/ARG KD cells with the highest level of 3D growth (**Figure 6A**), and extinguished active AKT in the cells (**Figure 6B**). In contrast, under standard culture conditions, the same doses of MK-2206 slowed but did not abolish cell proliferation (**Figure 6C**). Together with the previous data, these results indicate that one major consequence of Abl kinase deficiency can be to promote sustained AKT signaling, which can result in increased tumor cell growth on 3D matrix.

**Figure 7:**
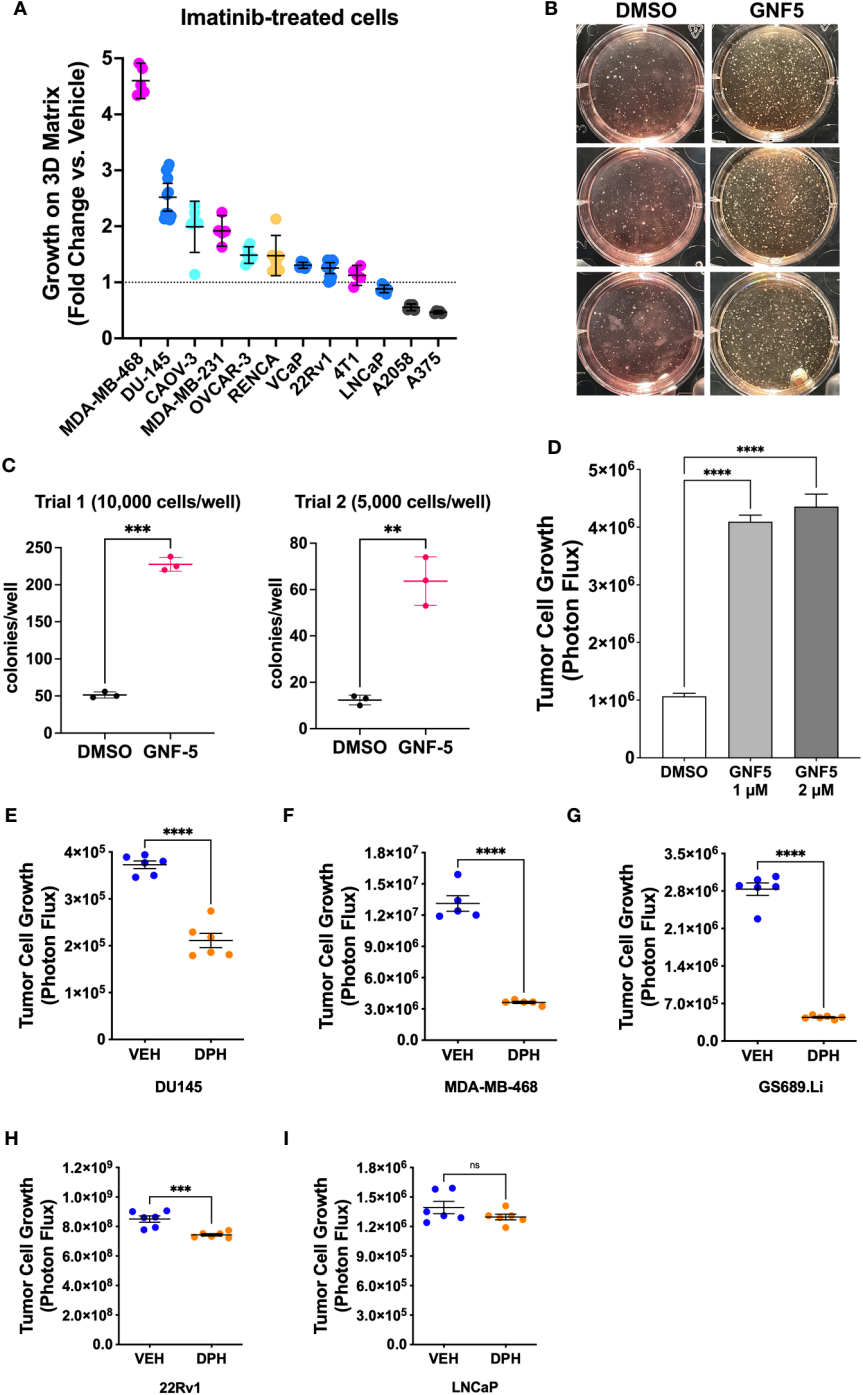
Abl kinase inhibition can promote malignant behavior in multiple cellular contexts. **(A)** Multiple tumor cell lines were grown in defined media on 3D collagen and treated with DMSO (vehicle) or 5 µM imatinib. For each cell line, data are plotted as fold change in 3D growth for imatinib-treated cells versus the DMSO vehicle treated cells. **(B)** Pre-malignant NIH-3T3 cells were plated in soft agar and cultured for 2 weeks in the presence or absence of DMSO vehicle or 2 µM GNF5. The photograph shows side-illuminated wells revealing a greatly increased number of colonies in the GNF5-treated cultures. **(C)** Quantification of two independent trials of NIH-3T3 soft agar assays with 10,000 or 5,000 cells plated per well in each of 3 wells per treatment. GNF5-treated cultures showed a 4-5 fold increase in the number of colonies formed, ***p<0.001, **p<0.01, unpaired t-test. **(D)** Growth of GS689.Li cells on 3D collagen treated with DMSO vehicle, 1 µM, or 2 µM GNF5. The GNF5-treated cells showed an ~4 fold increase in growth compared to DMSO-treated cells, ****p<0.0001, One-way ANOVA with Sidak multiple comparison test, α = 0.05. **(E–I)** Growth of different tumor cell types on 3D collagen treated with DMSO vehicle or 10 µM Abl kinase activator, DPH, ****p<0.0001, ***p = 0.001, n.s, not significant, unpaired t-test.

Lastly, to evaluate the generality of the link between Abl kinase signaling and tumor cell growth under low anchorage conditions, we tested the effect of imatinib on the 3D growth of 12 additional tumor cell lines. Imatinib enhanced the 3D growth of several of the cell lines, including prostate cancer cell line DU-145, breast cancer cell lines MDA-MB-468 and MDA-MB-231, and ovarian cancer cell line CAOV-3 (**Figure 7A**). We also evaluated the effect of Abl kinase inhibition on soft agar colony formation by NIH-3T3 fibroblasts. In this assay, it was necessary to use the highly specific, allosteric Abl kinase inhibitor, GNF5, because imatinib cross-inhibits PDGF receptors, upon which 3T3 cells rely (30). GNF5 promoted a striking 4-5-fold increase in colony formation in this assay (**Figures 7B, C**). Moreover, GNF5 also promoted growth on 3D collagen in our GS689.Li prostate cancer model (**Figure 7D**).

As an additional test of the role of Abl kinases in regulating 3D tumor cell growth, we investigated the effect of DPH, a selective Abl kinase activator, on three cell lines, DU-145, MDA-MB-468, and GS689.Li, which showed significantly enhanced 3D growth in response to imatinib, and two cell lines, 22Rv1 and LNCaP, which did not show a strong response to imatinib. We reasoned that if imatinib can promote 3D growth of specific tumor cell types via inhibition of Abl kinases, then DPH should suppress the 3D growth of those same tumor cell types, while tumor cell types that showed a more modest response to imatinib should also show a more modest response to DPH. Indeed, we found that DPH treatment substantially reduced the 3D growth of all three imatinib-responsive tumor cell types (**Figures 7E–G**). In contrast, 22Rv1 and LNCaP cells showed minimal response to DPH, with 22Rv1 cell growth being very modestly, albeit statistically significantly reduced, and LNCaP cell growth unaffected by DPH treatment (**Figures 7H, I**). Collectively, these experiments support the view that Abl kinase activity can function to suppress a malignant phenotype in variety of different settings, with some but not all cells responding to pharmacological Abl kinase manipulation.

## Discussion

A major finding of our study was the dramatic increase in the rate of tumor progression and metastasis of Abl kinase-deficient tumor cells, establishing that Abl kinases can function as suppressors of tumor progression and metastasis *in vivo*. Although inhibiting Abl kinase activity may be a potential therapeutic strategy for treating many types of solid tumors (14, 15), our new results add to a growing number of studies that point to the ability of Abl kinases to suppress cell motility, promote apoptosis, or negatively regulate cell proliferation and tumor growth in multiple other settings (16, 18–20, 31–35). Anti-tumor activity of Abl kinases has been linked to signaling by tumor suppressor EphB4 (20), p53-P21 signaling (16, 18, 35), and opposing the proto-oncogenic YAP1 transcription factor (34, 36). One caveat of our study is that we used RNAi to strongly suppress Abl kinase expression. It is possible that total loss of Abl expression, for example via CRISPR-mediated deletion, could produce a different outcome. Nevertheless, pharmacologically relevant concentrations of imatinib partially phenocopied several outcomes of RNAi depletion of Abl, both here and in our previous study (25). Therefore, strong, partial suppression of Abl kinases via RNAi can reveal phenotypes relevant to targeting Abl pharmacologically, which may also be expected to suppress but not totally eliminate all Abl signaling.

Our new data point to additional anti-tumor functions for Abl kinases that appear to be distinct from many of those that have previously been described. We find that Abl family kinase depletion results in sustained activation of AKT pathway signaling in our p53-null, PTEN-deficient prostate cancer model, in the absence of overt DNA damage and with no obvious impact on either signaling via the MAP kinase pathway, FAK/Src signaling, or the levels of the YAP1 transcription factor (**Figure S4**). Intriguingly, our data also support the view that, even in the context of PTEN deficiency, other factors controlled by Abl family kinase signaling can continue to restrain the full oncogenic capacity of the AKT pathway.

The mechanism by which Abl kinase deficiency can result in upregulation of the AKT pathway requires further study. Potential mechanisms include loss of an inhibitory interaction of Abl kinases with PI 3-kinase (37) or loss of Abl kinase stimulation of the PI(3,4,5)P3 lipid phosphatase, SHIP2 (38). Key open questions include (i) whether ABL nuclear localization is important for restraining tumor cell growth on 3D matrix, as originally described for Abl’s ability to suppress cellular transformation (39), (ii) which functional domains of the Abl kinases are required for suppressor functions, and (iii) what are the specific roles of ABL vs ARG when they do function as suppressors of tumor progression and metastasis? Regarding this last open question, our results have interesting overlaps and differences from those of a previous study, in which loss of ARG dramatically potentiated tumor cell growth but reduced spontaneous metastasis of MDA-MB-231 breast cancer cells (40).

Considering the magnitude of the increase in tumor progression and metastasis we observed in our prostate cancer model, it will be important to determine the cellular contexts in which Abl kinase inhibition may have paradoxical tumor promoting effects, since imatinib and other kinase inhibitors that cross-inhibit Abl are in clinical use. Our model of prostate cancer metastasis represents a mesenchymal, androgen-indifferent form of prostate cancer (26, 41) of the type that may emerge in advanced cases of heavily treated prostate cancer (42). Interestingly, in the TRAMP mouse model of prostate cancer, treatment with the Abl kinase inhibitor imatinib resulted in the selective outgrowth of androgen-indifferent, neuroendocrine-like tumors (43), and imatinib failed in prostate cancer clinical trials, where it was associated with reduced overall and progression-free survival (44). Moreover, a study of patients treated with imatinib for chronic myelogenous leukemia (CML) revealed a potential increase in incidence of secondary malignancies, including prostate cancer, compared to the general population (45). The increased incidence of secondary cancers in imatinib-treated patients was interpreted in this study as potentially due to a generally increased predisposition to cancer in CML patients. However, our data raise the possibility that Abl kinase inhibition could help to promote outgrowth of smoldering malignancies in specific cases, further underscoring the importance of understanding Abl kinase tumor suppressor functions at the molecular level.

While the cellular contexts in which Abl kinases can function as suppressors of progression and metastasis remain to be fully defined, in our panel of cell lines, androgen-driven VCaP, 22Rv1, and LNCaP prostate cancer cell lines did not respond strongly to Abl kinase inhibition, while androgen-indifferent DU145 prostate cancer cells, and basal-like MDA-MB-468 and MDA-MB-231 breast cancer cell lines showed a stronger increase in 3D growth upon Abl kinase inhibition (**Figure 7A**). Another possibility is that the complement of downstream Abl kinase substrates such as ABI1 and CRK may influence in which cell types Abl kinases may function as promoters versus suppressors of tumor progression. For example, ABI1 has been reported to function as a tumor suppressor in prostate cancer (21, 22, 24). Thus, determining the mechanism by which Abl kinases can restrain AKT pathway activation and metastatic progression may not only lead to identification of novel points of therapeutic intervention, but also provide valuable context for understanding under which circumstances Abl kinases may suppress versus promote malignant progression.

## Data availability statement

The original contributions presented in the study are included in the article/**Supplementary Material**. Further inquiries can be directed to the corresponding author.

## Ethics statement

Ethical approval was not required for the studies on humans in accordance with the local legislation and institutional requirements because only commercially available established cell lines were used. The animal study was approved by University of Iowa Institutional Animal Care and Use Committee. The study was conducted in accordance with the local legislation and institutional requirements.

## Author contributions

MM, MH, and CS contributed to conception and design of the study. MM, DM, AV, NJ, and DT performed experiments and analyzed data. MT contributed to the design and analysis of soft agar growth assays. MM wrote the first draft of the manuscript. CS helped write revised drafts of the manuscript. DL, MH, and CS edited the final version of the manuscript. JB contributed resources for the important for the collection of data and presented preliminary data at international meetings. All authors contributed to manuscript revision, read, and approved the submitted version.
