# Supplementary material for: Abl kinases can function as suppressors of tumor progression and metastasis
Source: Front Oncol. 2023 Sep 8;13:1241056. doi: 10.3389/fonc.2023.1241056 (PMC10514900; doi:10.3389/fonc.2023.1241056)
Supplement: Supplementary file 1 [file DataSheet_1.pdf]

Supplemental Figure Legends

Figure S1: Post-implantation analysis of ABL and ARG protein expression

Immunoblot analysis of ABL and ARG proteins in tumor cells recovered from primary tumors of mice bearing Abl family kinase-deficient (ABL KD, ARG KD, and ABL/ARG KD) and non-targeting (NT/NT) tumors for 3 mice/tumor type. Values beneath ABL and ARG protein bands represent blot intensity values normalized by an α–tubulin loading control. Values beneath α–tubulin protein bands represent raw blot intensity values.

Figure S2: Abl family kinase-deficient tumor cells display distinct morphological phenotypes during migration

The morphology of Abl family kinase deficient (ABL KD, ARG KD, and ABL/ARG KD) and non-targeting (NT/NT) tumor cells migrating on 2D collagen I was monitored via time-lapse microscopy and analyzed at t = 20 minutes. Time-lapse micrographs of **(A)** NT/NT, **(B)** ABL KD, **(C)** ARG KD, and **(D)** ABL/ARG KD mCRPC cell populations. Insets feature lamellae (L) and morphology of representative cells. Scale bar = 50 µm. Graphs depicting **(E)** the average aspect ratio and **(F)** average roundedness for Abl family kinase-deficient and non-targeting mCRPC cells. * and ** in E and F denote statistically significant p-values of < 0.05 and < 0.01, respectively. One-way ANOVA w/ Dunnett's multiple comparison, $n = 27$ cells, $\alpha = 0.05$.

Figure S3: Western blot validation of select RPPA differentially expressed antigens (DEAs)

(A-C) Tables of average normalized linear fold changes for selected groupings of DEAs: **(A)** AKT signaling components, **(B)** cell cycle factors, and **(D)** genes involved in lipogenesis and metabolism. **(D-F)** Immunoblot analysis of specific RPPA DEAs: **(D)** S6 Ribosomal Protein phosphorylated on serine 240/244 (pS6 S240/244), **(E)** Cyclin D3, **(F)** AMPKα phosphorylated

on threonine 172 (pAMKα T172), and their respective α–tubulin loading controls for Abl family kinase-deficient (ABL KD, ARG KD, and ABL/ARG KD) and non-targeting (NT/NT) tumor cells growing in 3D for 4 days.

Figure S4: The increased 3D growth of Abl family kinase-deficient mCRPC cells is not associated with activation of FAK, SRC, or ERK, or upregulation of YAP/TAZ

(A) Immunoblot analysis of FAK phosphorylated at tyrosine 397 (pFAK Y397), total FAK, SRC phosphorylated at tyrosine 416 (pSRC Y416), total SRC, ERK 1/2 phosphorylated at threonine 202 and tyrosine 204 (pERK T202/Y204), and total ERK 1/2 for Abl family kinase deficient (ABL KD, ARG KD, and ABL/ARG KD) and non-targeting (NT/NT) tumor cells growing under 3D conditions for 4 days. **(B)** Immunoblot analysis of YAP/TAZ and tubulin loading control for Abl family kinase deficient (ABL KD, ARG KD, and ABL/ARG KD) and non-targeting (NT/NT) tumor cells growing under 3D conditions for 4 days.

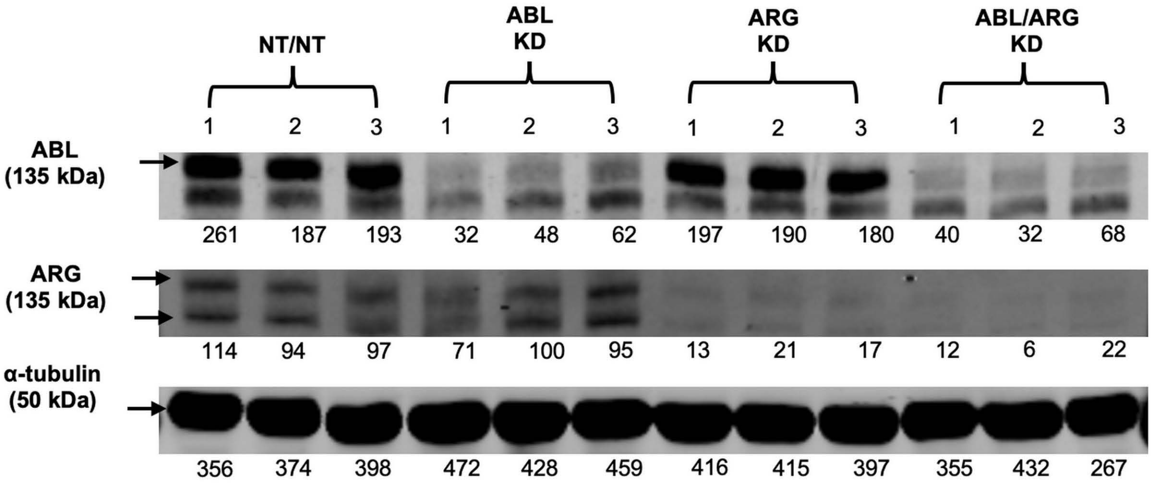
NT/NT
ABL KD
ARG KD
ABL/ARG KD
1 2 3 1 2 3 1 2 3 1 2 3
ABL (135 kDa)
261 187 193 32 48 62 197 190 180 40 32 68
ARG (135 kDa)
114 94 97 71 100 95 13 21 17 12 6 22
α-tubulin (50 kDa)
356 374 398 472 428 459 416 415 397 355 432 267

Figure S1

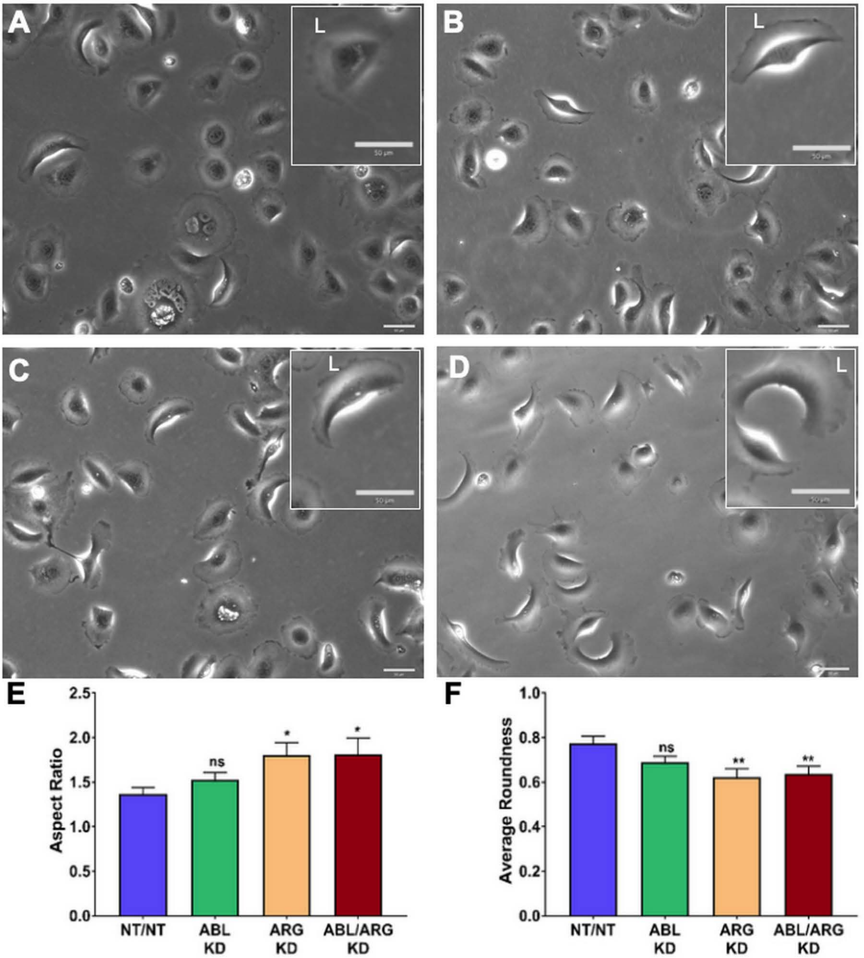

$$\text{Roundness} = 4\times\frac{[\text{Area}]}{\pi\times[\text{Major Axis}]^2}$$

Figure S2

A

Canonical AKT Signaling Components	
Gene Name	Average RPPA Fold Change
4E-BP1 pS65	1.3 ↑
AKT pS473	1.5 ↑
GSK3 α/β pS21/S9	1.2 ↑
PDK1	1.2 ↑
pS6 S240/244	1.4 ↑

B

Cell Cycle	
Gene Name	Average RPPA Fold Change
CDC25c	1.3 ↑
Cyclin B1	1.5 ↑
Cyclin D3	1.3 ↑
p21	1.2 ↑
Rb pS807/S811	1.2 ↑

C

Lipogenesis and Metabolism	
Gene Name	Average RPPA Fold Change
ACC1	1.6 ↑
AMPKα pT172	0.7 ↓
FASN	1.5 ↑
G6PD	1.5 ↑
PAICS	1.3 ↑

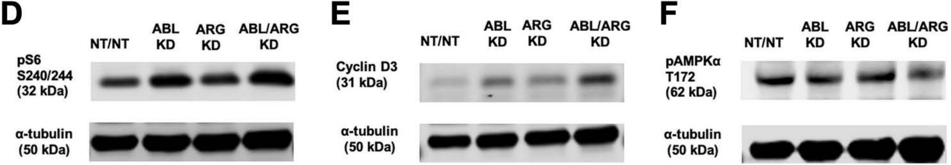

Figure S3

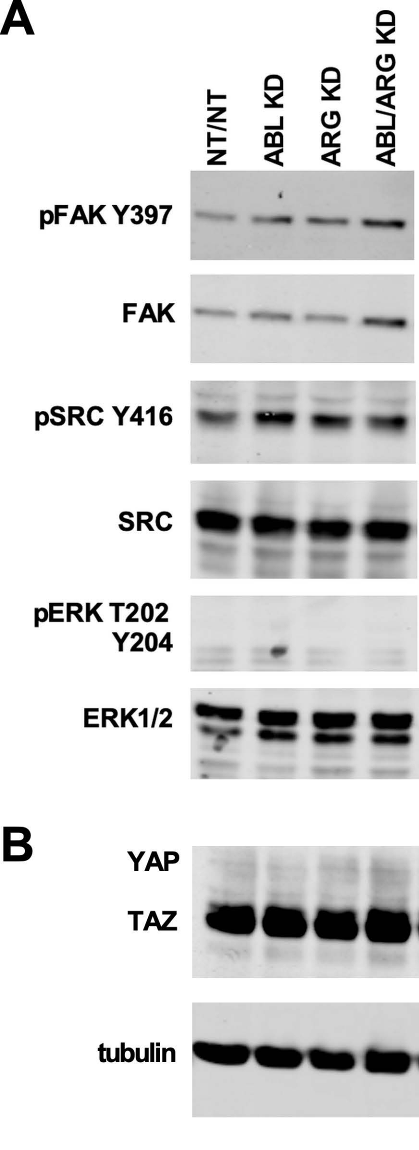
A
NT/NT
ABL KD
ARG KD
ABL/ARG KD
pFAK Y397
FAK
pSRC Y416
SRC
pERK T202 Y204
ERK1/2
B
YAP
TAZ
tubulin

Figure S4
